# TP53 mediated miR‐3647‐5p prevents progression of cervical carcinoma by targeting AGR2

**DOI:** 10.1002/cam4.2507

**Published:** 2019-08-22

**Authors:** Ronghua Liu, Min Qian, Ting Zhou, Pengfei Cui

**Affiliations:** ^1^ Department of Obstetrics and Gynecology Tongji Hospital Tongji Medical College Huazhong University of Science and Technology Wuhan Hubei China; ^2^ Department of Obstetrics and Gynecology General Hospital of Eastern Theater Command Nanjing Jiangsu China

**Keywords:** AGR2, cervical cancer, miR‐3647‐5p, TP53

## Abstract

Previous studies have shown that miRNAs involved in a number of biological processes, such as cell growth, development, differentiation, and apoptosis. The dysregulation of miRNA expression is associated with various diseases, including cervical cancer. However, the involvement of miR‐3647‐5p in the progression of tumors is unclear. In this study, we confirmed that miR‐3647‐5p was down‐regulated during cervical carcinogenesis and development, which was positively correlated with the prognosis of patients with cervical cancer. In addition, our study showed that miR‐3647‐5p can inhibit the proliferation of cervical cancer cells and promote apoptosis, suggesting that miR‐3647‐5p is involved in the development of cervical cancer as a tumor suppressor gene. Furthermore, we found that transcription factor TP53 could promote the expression of miR‐3647‐5p, suggesting that the dysfunction of miR‐3647‐5p in cervical cancer may be related to TP53. In addition, we also found that miR‐3647‐5p can inhibit the proliferation of cervical cancer cells and promote apoptosis by targeting AGR2. In summary, our research reveals that transcription factor TP53 promotes the expression of miR‐3647‐5p, while up‐regulated miR‐3647‐5p targets AGR2, inhibiting cervical cancer cell proliferation and promoting apoptosis. Our study reveals the mechanism of TP53/miR‐3647‐5p/AGR2 axis in cervical cancer, which may be useful for targeted therapy of cervical cancer.

## Graphical abstract

Our research reveals that TP53 acts as a transcription factor to promote the expression ofmiR‐3647‐5p, while up‐regulated miR‐3647‐5p targets AGR2, inhibiting cervical cancer cell proliferation and promoting apoptosis.

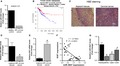

## 1 INTRODUCTION

Cervical cancer is a common malignant cancer in women. Approximately 527 600 cases of cervical cancer are diagnosed every year around the world, with approximately 265 700 deaths.^1^ Epidemiological statistical analysis shows that human papillomavirus (HPV) infection is one of the most important risk factors for cervical cancer, followed by abortion, uterine cervicitis, etc.^2^ In recent years, with the improvement of diagnosis and treatment, the survival time and quality of life of cervical cancer patients have been greatly improved. However, the 5‐year survival rate of patients with cervical cancer in the lowly developed country is less than 20%, while exceed 65% in the highly developed country.^3^ Cervical cancer is still one of the serious threats to women's health. Therefore, it is still urgent to further explore the pathogenesis of cervical cancer and to find new strategies for targeted therapy.

Noncoding RNA, as the name suggests, refers to RNA that cannot be translated into a protein. It can be divided into two types: housekeeping noncoding RNA and Regulatory noncoding RNA. The latter includes short‐chain (including small interfering RNA [siRNA], microRNA [miRNA], Piwi‐interactiing RNA) and long‐length (long noncoding RNA, lncRNA) noncoding RNA.^4^ MicroRNAs are a large class of single‐chain, highly conserved, noncoding, short‐stranded RNAs composed of 19‐24 nucleotides, widely found in many eukaryotes and viruses, 50% of which are localized to chromosomes region prone to structural changes.^5^ miRNA directly targets mRNA and exerts posttranscriptional regulation, and binds to the target gene 3′untranslated regions (3′UTR) through the seed sequence. When the miRNA is fully complementary to the target gene, it directly causes cleavage of the mRNA; when it is incompletely complementary to the target gene, it represses the translation of the gene.^6^ Previous studies have shown that miRNAs involve in a number of biological processes, such as cell growth, development, differentiation, apoptosis, and endocrine balance. The dysregulation of miRNA expression is associated with various diseases, including tumors, cardiovascular diseases, autoimmune diseases and neurodegenerative diseases.^7^ Dysfunction of miRNA has been reported to participate in tumorigenesis and progression by promoting uncontrolled proliferation, increasing survival rate, inhibiting differentiation, and promoting cancer cells metastasis.^8^ Calin et al found that miR‐15 and miR‐16 play a role as tumor suppressor genes, which is the earliest report on miRNAs and tumors.^9^ Since then, studies have reported abnormal expression of miRNAs in various human tumors, including cervical cancer, ovarian cancer, breast cancer, lung cancer, kidney cancer, colon cancer, and bladder cancer.^10^ Considering that miRNA plays an important role in tumors, more research is beginning to explore the application of miRNAs as targets for tumor treatment.^11^ At present, miRNA‐based tumor targeted therapy is still in the preclinical research stage, so it is especially important to find key miRNAs in the process of tumor development. In the previous study, we revealed that miR‐3647‐5p was significantly down‐regulated in HPV‐related cervical cancer using bioinformatics analysis, suggesting that it may play an important role in the development of cervical cancer. However, there is no research to investigate the relationship between miR‐3647‐5p and tumorigenesis.

In this study, we found that the expression of miR‐3647‐5p is down‐regulated in the development of cervical cancer. In cervical cancer cells, we reveal that TP53‐mediated upregulation of miR‐3647‐5p can inhibit the proliferation of cancer cells and promote cell apoptosis by targeting AGR2. Our study indicates that miR‐3647‐5p plays an important role in the development of cervical cancer as a tumor suppressor gene.

## 2 MATERIALS AND METHODS

### 2.1 Cell culture and cervical tissues

Normal cervical epithelial cells HCerEpiC, and cervical cancer cells SiHa, Caski and C33A were bought from the Cell Bank of Type Culture Collection of the Chinese Academy of Sciences (Shanghai, China). The cells were cultured in Dulbecco's modified eagle medium (HyClone), containing 10% FBS (fetal bovine serum) (Gibco), penicillin (100 U/mL) and streptomycin (100 mg/mL) at 37°C, 5% CO_2_.

Fifteen cases of cervical cancer tissues and paracancerous tissues were from the Department of Obstetrics and Gynecology, Tongji Hospital, Tongji Medical College, Huazhong University of Science and Technology. All patients were informed and agreed with the application of tissues. Our study was approved by the Ethics Board of Tongji Hospital, Tongji Medical College, Huazhong University of Science and Technology and complied with all relevant principles of the Declaration of Helsinki.

### 2.2 Plasmid, miRNA and cell transfection

Overexpression plasmids of AGR2 and TP53 were constructed and purchased from Sangon Company. The promoter of miR‐3647‐5p was cloned to pGL3‐Basic vector to generate luciferase reporter plasmid, and the mutant luciferase reporter plasmid was generated by mutating G to C and C to G. The miR‐3647‐5p mimic and inhibitor were purchased from Genepharma Company; the mimic sequence was CCUGAAGUGAUGAUUCACAUUCA, and the inhibitor sequence was CCGTGGTAGATGCCTGTGGTA with the control miRNA sequence being UCUCCGAACGUGUCACGUU. The cells were transfected using Lipofectamine 2000 reagent (Invitrogen), according to the manufacturer's instruction.

### 2.3 Reverse transcription and quantitative polymerase chain reaction

Total RNA of cells and tissues was extracted with Trizol reagent (Invitrogen), according to the manufacturer's instructions, and then total RNA was reverse transcribed into cDNA using a reverse transcription kit (Thermo fisher). The generated cDNA was used to detect the expression of target genes using a SYBR Premix Ex Taq II kit (Takara). The RT primer for miR‐3647‐5p was GTCGTATCCAGTGCAGGGTCCGAGGTATTCGCACTGGATACGACTGAATG. The RT primer for U6 was GTCGTATCCAGTGCAGGGTCCGAGGTATTCGCACTGGATACGACAAATATG.The primers for qPCR included miR‐3647‐5p (5′‐CCTGAAGTGATGATTCA‐3′ and 5′‐ACTGTACTGGAAGATGGAC ‐3′), U6 (5′‐CTCGCTTCGGCAGCACA‐3′ and 5′‐AACGCTTCACGAATTTGCGT‐3′), Ki‐67 (5′‐TCGTCCCAGTGGAAGAGTTG‐3′ and 5′‐GTCTCGTGGGCCACATTTTC‐3′), cyclin A1 (5′‐ATAACGACGGGAAGAGCGGG‐3′ and 5′‐CTCCATCCCAAGTGACGAGC‐3′), cyclin B1 (5′‐CAGGTTGTTGCAGGAGACCA‐3′ and 5′‐AACATGGCAGTGACACCAAC‐3′, cytochrome C (Cyt C) (5′‐ATGAGTAATAATTGGCCACTGCC‐3′ and 5′‐AATCAGGACTGCCCAACAAA‐3′, Bax (5′‐GGGTTGTCGCCCTTTTCTAC‐3′ and 5′‐AGTCGCTTCAGTGACTCGG‐3′), AGR2 (5′‐GACTCACACAAGGCAGGTGG‐3′ and 5′‐ATGAGTTGGTCACCCCAACC‐3′), CYP4B1 (5′‐CCTCTCCCTGAGCTTCTCCT‐3′ and 5′‐CGAGGGCATGTCCAAAAAGC‐3′), PIGR (5′‐CTCCTAGAGAGGCAGGGGTT‐3′ and 5′‐GAGCACGAAGAGCAGCATTG‐3′), PIGR (5′‐CTCCTAGAGAGGCAGGGGTT‐3′ and 5′‐GAGCACGAAGAGCAGCATTG‐3′), MUC‐13 (5′‐CTATTGTGGGCACCATCGCT‐3′ and 5′‐GAAAGACGCTCCCTTCTGCT‐3′), CTCFL (5′‐CCTCCTCCCTTCCTCATCCA‐3′ and 5′‐ACTCCGTCTTTGGCTTGTGG‐3′), GABRP (5′‐GATGAGGAGAACCCTGGGGA‐3′ and 5′‐CACCAAGGGGCTGAAGTAGG‐3′), CYP4X1 (5′‐TTCTTCCCGCGAGTCAGAAG‐3′ and 5′‐CAGAACACGAACGCCAGGTA‐3′), CTSE (5′‐CTCTGGGTCCCCTCTGTGTA‐3′ and 5′‐GCTCCAATGATCCCGGACAA‐3′), KRT7 (5′‐CATCGAGATCGCCACCTACC‐3′ and 5′‐TGGAGAAGCTCAGGGCATTG‐3′), RARRES1 (5′‐CGGCGCTTCACTTCTTCAAC‐3′ and 5′‐CTCTGGGTTGTAGCGCTCTG‐3′), GAPDH (5′‐TCGGAGTCAACGGATTTGGT‐3′ and 5′‐TTCCCGTTCTCAGCCTTGAC‐3′).

### 2.4 Western blotting

The protein was extracted from cells using radio immunoprecipitation assay lysis buffer. Bicinchoninic acid Protein Assay Kit (Beyotime Biotechnology) was used to measure protein concentration. The experimental process of western blotting is conventional and the same as the previous article.^12^ The antibodies used in this study include cyclin A1, cyclin B1, Cyt C, Bax, TP53, AGR2 and α‐Tubulin, which were all purchased from Cell Signaling Technology.

### 2.5 Clone formation assay and CCK‐8 assay

The proliferation ability of cells was detected using colony formation assay and cell counting kit‐8 (CCK‐8) assay. For the colony formation assay, in brief, cells of each group were seeded in a six‐well cell culture plate, and after about 1 week, the cells were stained and counted with crystal violet using microscopy.

For the CCK‐8 assay, cells of each group were seeded in 96‐well cell culture plate, and the proliferation ability of cells was assessed using CCK‐8 kit (Dojindo) on a microplate reader (BD company), according to the manufacturer's protocol.

### 2.6 Flow cytometry

Flow cytometry was used to detect cell apoptosis using an Annexin V‐FITC/PI Apoptosis Detection Kit (BD). According to the manufacturer's protocol, and the cells were labeled with Annexin V‐FITC/PI and analyzed using flow cytometry.

### 2.7 Luciferase assay

Cells of each group were transfected with report gene plasmids using Lipofectamine 2000. After transfection for 48 hours, the relative luciferase value was detected with a GloMax 20/20 Luminometer (Promega) using Dual‐Luciferase reporter Assay Kit (Promega).

### 2.8 Statistical analysis

Statistical analysis was performed using SPSS 13.0 software. The data were expressed as mean ± standard deviation (SD). The statistical analysis between groups was performed using one‐way ANOVA. The correlation between miR‐3647‐5p and Ki67 expression was analyzed using Pearson Correlation Coefficient. *P* < .05 was considered statistically significant.

## 3 RESULTS

### 3.1 miR‐3647‐5p is down‐regulated in cervical cancer tissues and cells, and is positively correlated with the prognosis of cervical cancer patients

**Figure 1:**
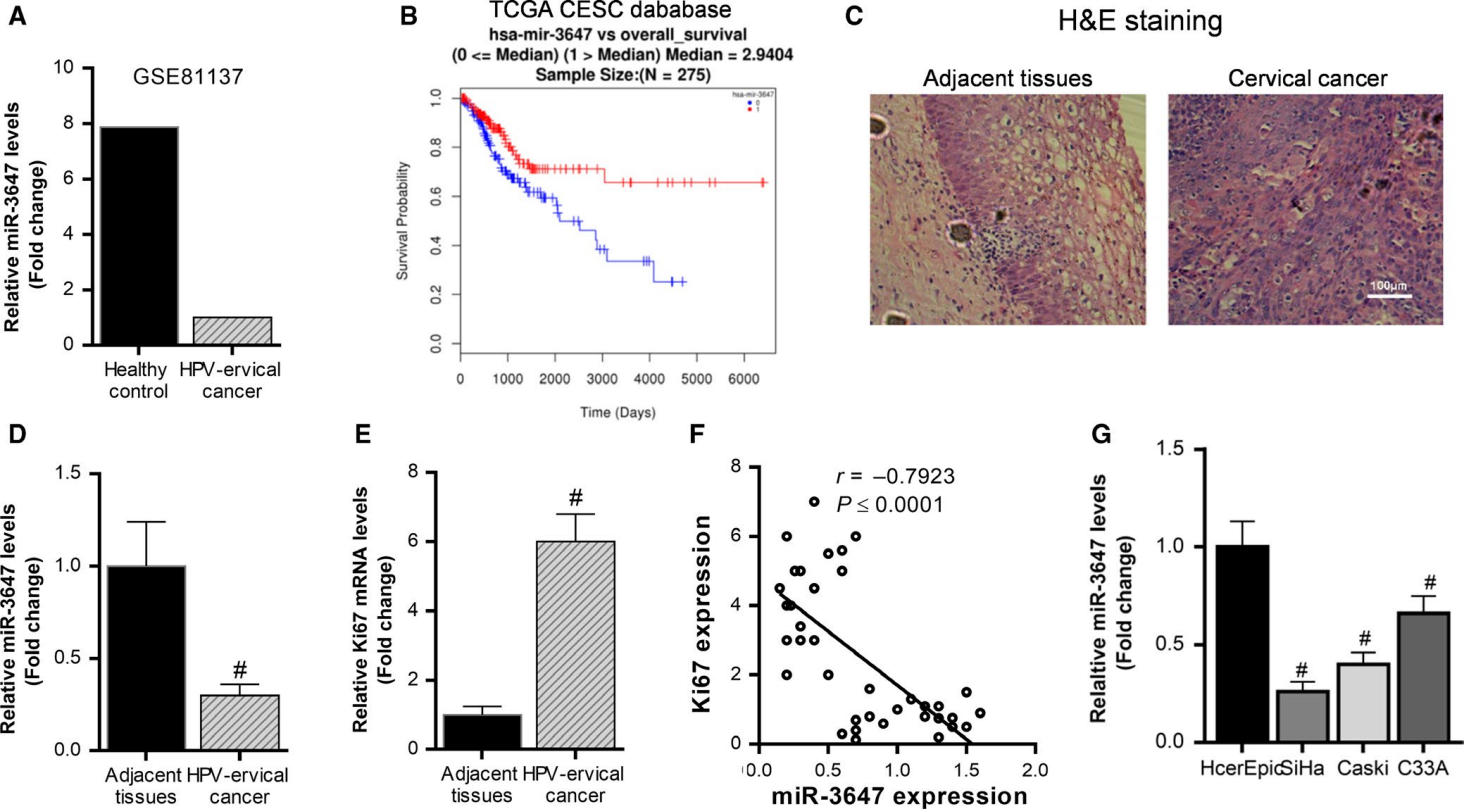
The expression of miR‐3647‐5p is decreased in cervical cancer tissues and cells, which is positively correlated with the prognosis of cervical cancer patients. A, Transcriptome sequencing (GSE81137) was used to analyze the expression levels of miR‐3647‐5p in cervical cancer tissues and normal tissues. B, Analysis of the association between miR‐3647‐5p and prognosis in patients with cervical cancer using the TCGA CESC database. Cervical cancer tissues and adjacent tissues were collected, (C) pathologically diagnosed using hematoxylin‐eosin staining, (D, E) and the expression levels of miR‐3647‐5p and Ki67 were detected using reverse transcription and quantitative polymerase chain reaction (RT‐qPCR), ^#^ *P* < .05, n = 15. F, Analysis of the correlation between miR‐3647‐5p and Ki67 expression in cervical cancer tissues. G, The expression of miR‐3647‐5p in normal cervical epithelial cells and cervical cancer cells was detected using RT‐qPCR, ^#^ *P* < .05, compared to HcerEpic cells

In order to distinguish the expression of miR‐3647‐5p in cervical cancer tissues and normal tissues, we first analyzed the transcriptome sequencing data, and found that miR‐3647‐5p was significantly reduced in HPV‐associated cervical cancer tissues relative to normal tissues (Figure 1A). Then, through the TCGA CESC database analysis, we found that the expression level of miR‐3647‐5p was positively correlated with the overall survival of patients with cervical cancer (Figure 1B). To further validate the differential expression of miR‐3647‐5p, we collected 15 cases of cervical cancer tissues and paracancerous tissues (Figure 1C). The reverse transcription and quantitative polymerase chain reaction (RT‐qPCR) results showed that miR‐3647‐5p in cervical cancer tissues was significantly down‐regulated relative to adjacent tissues (Figure 1D). At the same time, we also examined the mRNA expression level of Ki67, a marker of cell proliferation, and the results showed that Ki67 in cervical cancer tissues was significantly up‐regulated relative to adjacent tissues (Figure 1E). Moreover, miR‐3647‐5p was significantly negatively correlated with the expression of Ki67 (Figure 1F). In addition, we also detected the expression levels of miR‐3647‐5p in normal cervical epithelial cells HCerEpiC, and cervical cancer cells SiHa, Caski and C33A. The results showed that miR‐3647‐5p expression in SiHa, Caski and C33A was significantly lower than in HCerEpiC cells (Figure 1G). These results indicate that miR‐3647‐5p may be a tumor suppressor gene in cervical cancer.

### 3.2 miR‐3647‐5p can suppress the proliferation of cervical cancer cells and promote cell apoptosis

**Figure 2:**
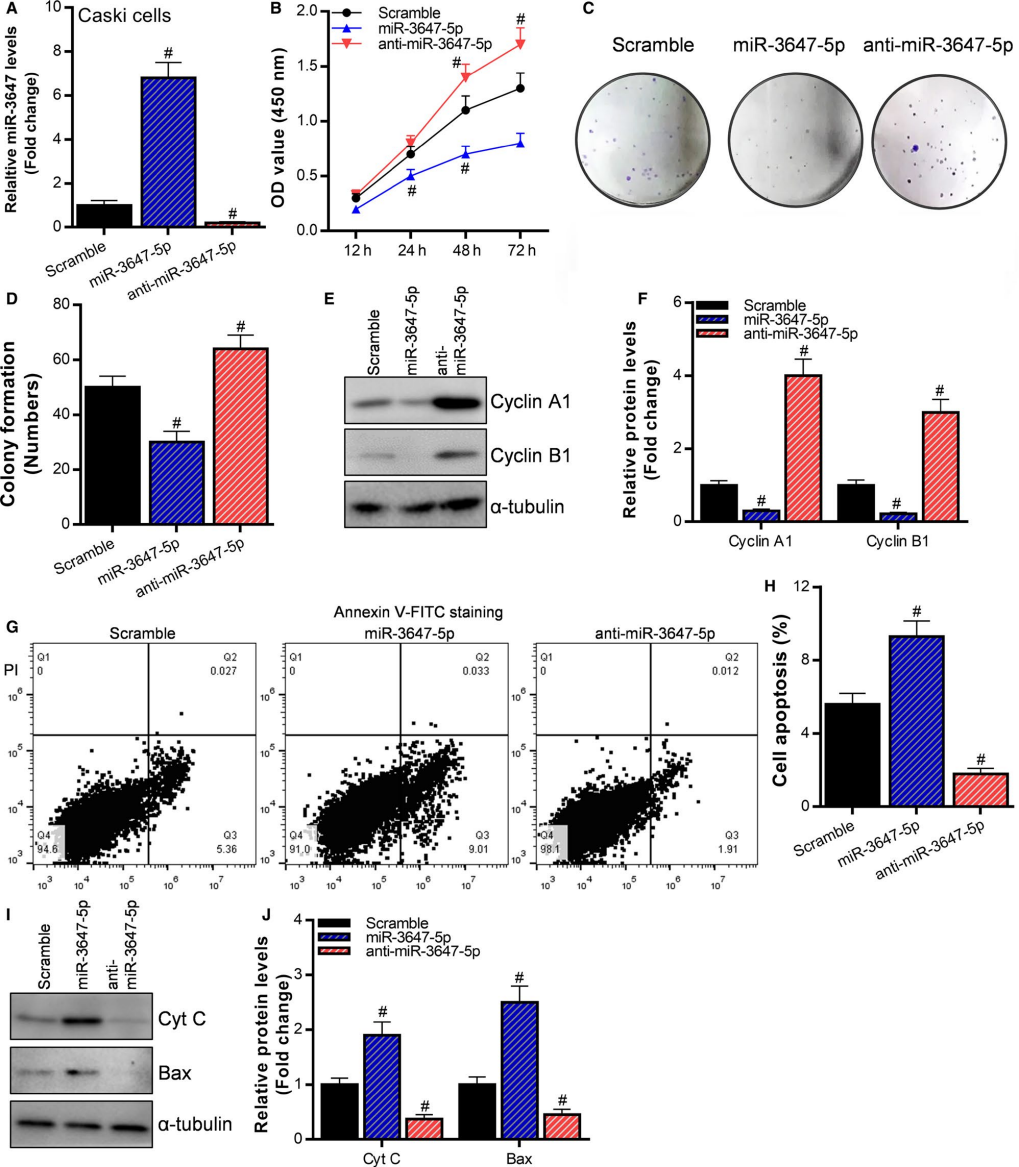
miR‐3647‐5p can inhibit the proliferation of cervical cancer cells and promote apoptosis. A, miR‐3647‐5p mimic (miR‐3647‐5p) and miR‐3647‐5p inhibitor (anti‐miR‐3647‐5p) were transfected into Caski cells, the effect of miR‐3647‐5p overexpression and knockdown was detected using reverse transcription and quantitative polymerase chain reaction, ^#^ *P* < .05, compared to the Scramble group. B, The effect of miR‐3647‐5p overexpression and knockdown on the proliferation of Caski cells was detected using CCK‐8 assay, ^#^ *P* < .05, compared with the Scramble group. (C, D) Clone formation assay was used to examine the effect of miR‐3647‐5p overexpression and knockdown on the proliferation of Caski cells, ^#^ *P* < .05, compared to the Scramble group. (E, F) Western blot was used to detect the effect of miR‐3647‐5p overexpression and knockdown on proliferation‐related protein expression in Caski cells, ^#^ *P* < .05, compared to the Scramble group. (G, H) Flow cytometry was used to detect the effect of miR‐3647‐5p overexpression and knockdown on cell apoptosis in Caski cells, ^#^ *P* < .05, compared to the Scramble group. (I, J) Western blot was used to detect the effect of miR‐3647‐5p on the expression of apoptosis‐associated proteins in Caski cells, ^#^ *P* < .05, compared to the Scramble group. Cyt C, cytochrome C; OD, oligomerization domain

We have confirmed that miR‐3647‐5p is down‐regulated during cervical carcinogenesis and is negatively correlated with the expression of Ki67. What role does miR‐3647‐5p play in cervical cancer? We purchased miR‐3647‐5p mimic (miR‐3647‐5p) and inhibitor (anti‐miR‐3647‐5p) and then transfected into cervical cancer Caski (Figure 2A). CCK‐8 assay and clone formation assay were used to detect the proliferative capacity of cervical cancer cells. The results of CCK‐8 assay showed that miR‐3647‐5p can significantly inhibit the proliferation of Caski cells, while anti‐miR‐3647‐5p can significantly enhance the ability of cell proliferation (Figure 2B). The results of the colony formation experiments were consistent with the CCK‐8 assay (Figure 2C,D). Both cyclin A1 and cyclin B1 are members of the cyclin family. They promote and coordinate cell cycle progression by binding to cyclin‐dependent kinases (CDKs) and modulating the enzymatic activity of CDKs.^13^ Therefore, upregulation of cyclin A1 and cyclin B1 can promote cell proliferation. In Caski cells, miR‐3647‐5p significantly up‐regulated the protein expression levels of cyclin A1 and cyclin B1, while anti‐miR‐3647‐5p significantly inhibited the expression of cyclin A1 and cyclin B1 (Figure 2E,F). Next, we examined the effect of miR‐3647‐5p on the apoptosis of Caski cells using flow cytometry. The results showed that miR‐3647‐5p can significantly promote the apoptosis of Caski cells, while anti‐miR‐3647‐5p significantly inhibits the apoptosis of Caski cells (Figure 2G,H). Cytochrome C and Bax are both apoptosis‐related proteins that promote apoptosis. In Caski cells, miR‐3647‐5p significantly up‐regulated the protein expression levels of Cyt C and Bax, while anti‐miR‐3647‐5p significantly inhibited the expression of Cyt C and Bax (Figure 2I,J). These results indicate that miR‐3647‐5p can inhibit the proliferation of cervical cancer cells and promote apoptosis.

### 3.3 AGR2 is the target gene of miR‐3647‐5p

**Figure 3:**
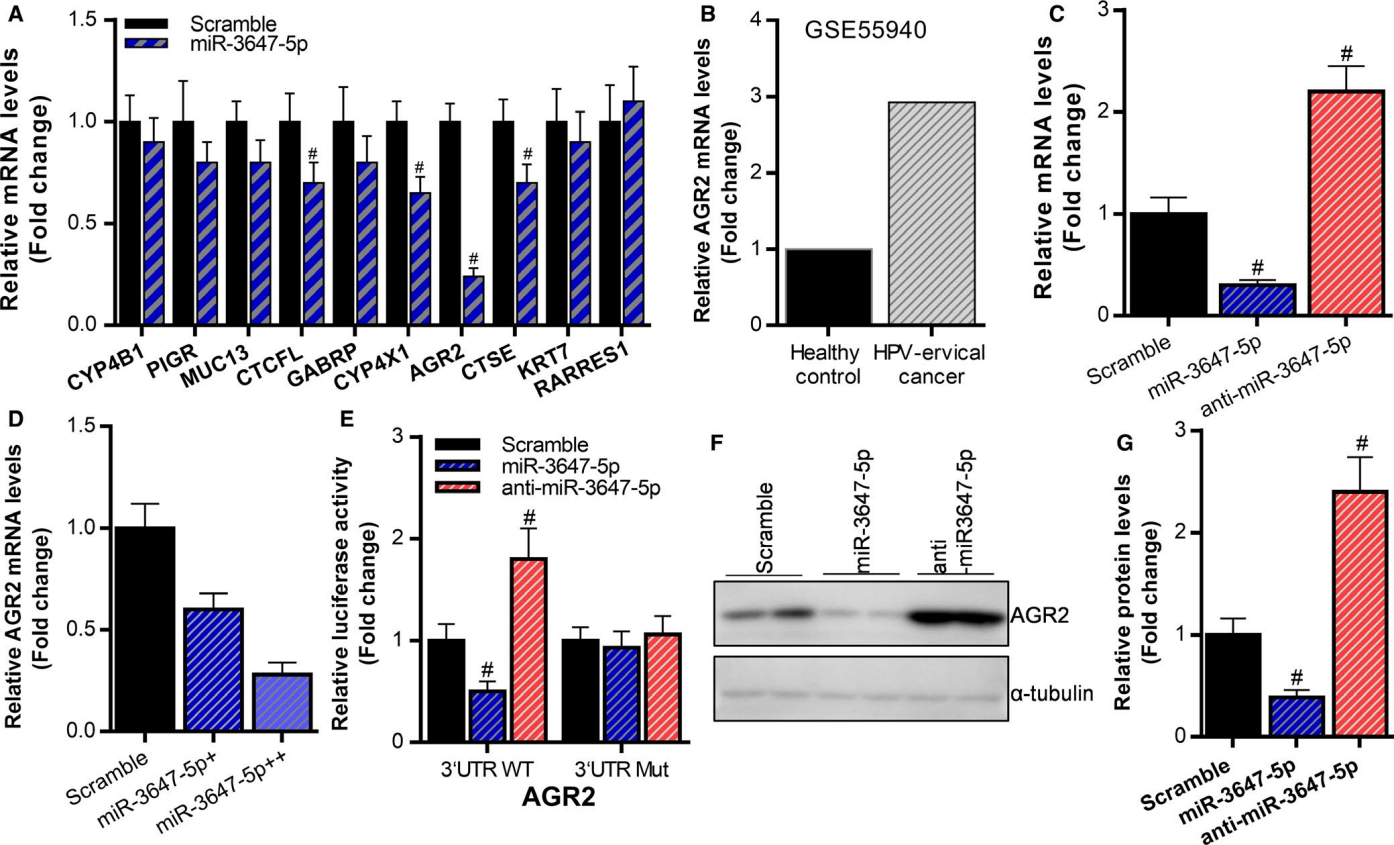
AGR2 is the target gene of miR‐3647‐5p. A, The effect of miR‐3647‐5p on the expression of potential target genes was detected using reverse transcription and quantitative polymerase chain reaction (RT‐qPCR), ^#^ *P* < .05, compared to the Scramble group. B, Transcriptome sequencing (GSE81137) was used to analyze the expression of AGR2 in cervical cancer tissues and normal tissues. C, The effect of miR‐3647‐5p on mRNA expression of AGR2 was detected using RT‐qPCR, ^#^ *P* < .05, compared to the Scramble group. D, The effect of miR‐3647‐5p on the mRNA expression level of AGR2 at different transfection concentrations was detected using RT‐qPCR, ^#^ *P* < .05, compared to the Scramble group. (E, F) Western blot was used to detect the effect of miR‐3647‐5p overexpression or knockdown on the protein level of AGR2, ^#^ *P* < .05, compared to the Scramble group. G, Luciferase assay was used to detect the effect of miR‐3647‐5p overexpression or knockdown on the activity of AGR2 3'untranslated regions (3′UTR) or binding site mutations reporter plasmid, ^#^ *P* < .05, compared to the Scramble group

We have confirmed that miR‐3647‐5p is a tumor suppressor gene, but the function of miR‐3647‐5p in cervical cancer is still unclear. MiRNAs usually regulate the expression of target genes by scilencing mRNA, thereby exerting biological functions. Bioinformatics analysis revealed that potential target genes for miR‐3647‐5p include CYP4B1, PIGR, MUC13, CTCFL, GABRP, CYP4X1, AGR2, CTSE, KRT7, and RARRES1. Then, we transfected miR‐3647‐5p mimic (miR‐3647‐5p) and then detected the expression levels of these potential target genes using RT‐qPCR. The results showed that miR‐3647‐5p can significantly inhibit CTCFL, CYP4X1, AGR2 and CTSE, in which miR‐3647‐5p has the most obvious inhibitory effect on AGR2 (Figure 3A). Therefore, we further verified whether AGR2 is a target gene of miR‐3647‐5p. The correlation between AGR2 and cervical cancer is still unclear. Therefore, we analyzed the transcriptome sequencing by bioinformatics, and found that compared to normal cervical tissues, the expression of AGR2 were significantly upregulated in cervical cancer tissues (Figure 3B). In Caski cells, miR‐3647‐5p significantly inhibited AGR2 mRNA expression, while anti‐miR‐3647‐5p significantly up‐regulated AGR2 mRNA expression (Figure 3C). In addition, the inhibitory effect of miR‐3647‐5p on AGR2 was positively correlated with the amount of transfection (Figure 3D). The results of western blot also showed that miR‐3647‐5p can significantly inhibit the expression of AGR2, while anti‐miR‐3647‐5p can significantly up‐regulate AGR2 (Figure 3E,F). In addition, miR‐3647‐5p significantly inhibited the reporter gene activity of AGR2 3′UTR, while anti‐miR‐3647‐5p significantly enhanced the reporter gene activity of AGR2 3′UTR, but miR‐3647‐5p and anti‐miR‐3647‐5p had no significant effect on the 3′UTR reporter gene activity in which the binding sequence was mutated (Figure 3G). These results indicate that AGR2 is a target gene of miR‐3647‐5p.

### 3.4 miR‐3647‐5p inhibits the proliferation of cervical cancer cells and promotes cell apoptosis by targeting AGR2

**Figure 4:**
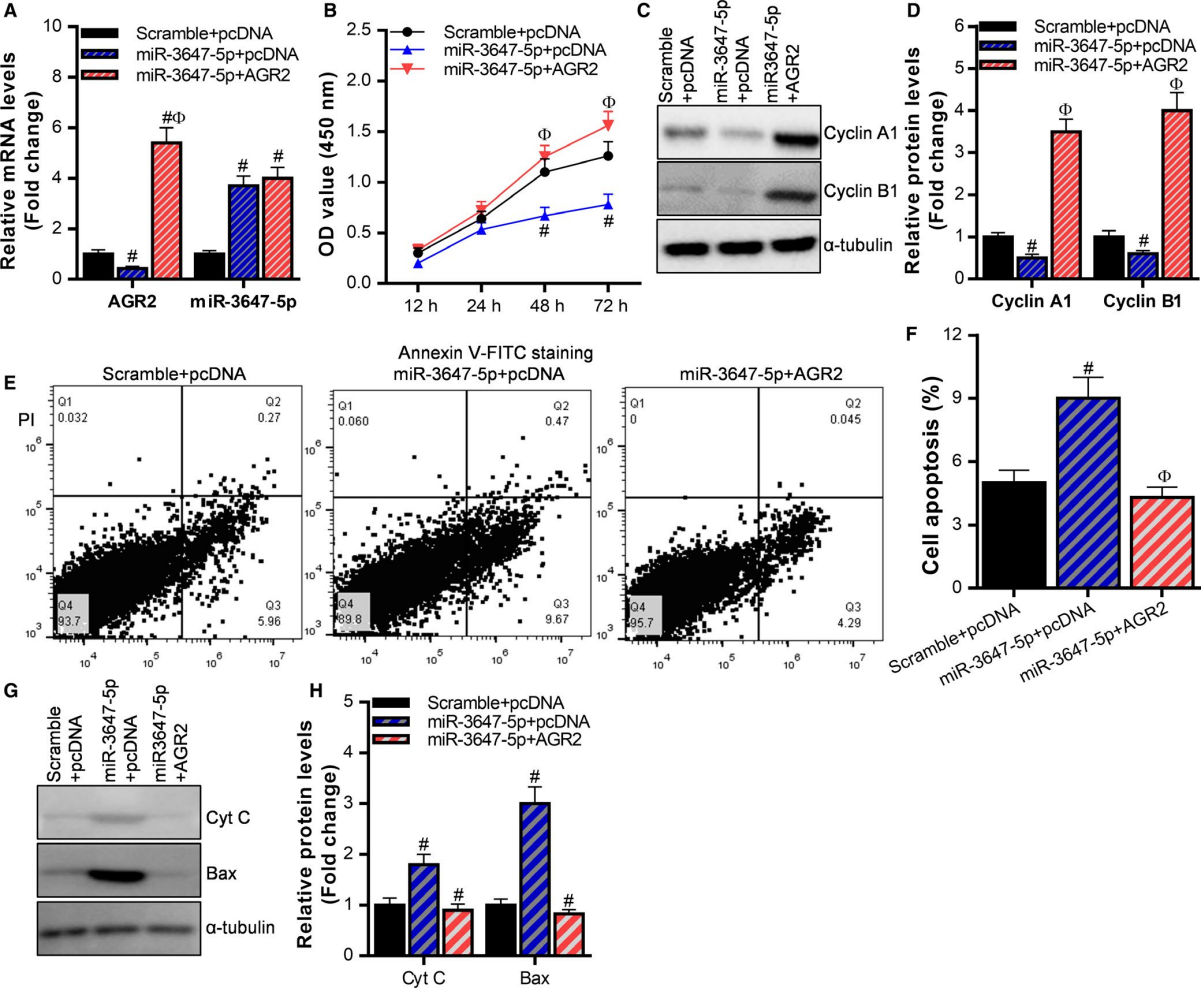
miR‐3647‐5p inhibits the proliferation of cervical cancer cells and promotes cell apoptosis by targeting AGR2. A, The expression levels of AGR2 and miR‐3647‐5p in each group were detected using reverse transcription and quantitative polymerase chain reaction, ^#^ *P* < .05, relative to the Scramble + pcDNA group, ^Ф^ *P* < .05, relative to the miR‐3647‐5p + pcDNA group. B, CCK‐8 assay was used to detect the proliferation of cells in each treatment group, ^#^ *P* < .05, relative to the Scramble + pcDNA group, ^Ф^ *P* < .05, relative to the miR‐3647‐5p + pcDNA group. (C, D) Western blot was used to detect the expression of proliferation‐related proteins in each treatment group, ^#^ *P* < .05, relative to the Scramble + pcDNA group, ^Ф^ *P* < 0.05, relative to the miR‐3647‐5p + pcDNA group. (E, F) Flow cytometry was used to detect cell apoptosis in each treatment group, ^#^ *P* < .05, relative to the Scramble + pcDNA group, ^Ф^ *P* < .05, relative to the miR‐3647‐5p + pcDNA group. (G, H) Western blot was used to detect the expression of apoptosis‐related protein in each treatment group, ^#^ *P* < .05, relative to Scramble + pcDNA group, ^Ф^ *P* < .05, relative to miR‐3647‐5p + pcDNA group

In general, miRNAs exert biological functions by regulating the expression of target genes. So is the effect of miR‐3647‐5p on cervical cancer cells Caski cells related to AGR2? In cervical cancer Caski cells, we observed the effect on cell proliferation and apoptosis by transfecting miR‐3647‐5p mimic (miR‐3647‐5p) with AGR2 overexpression (Figure 4A). It was shown that miR‐3647‐5p can significantly inhibit the proliferation of Caski cells, while overexpression of AGR2 can reverse the suppressive effect of miR‐3647‐5p on the cell proliferation (Figure 4B). Western blot analysis also showed that AGR2 overexpression can reverse the suppressive effect of miR‐3647‐5p on the expression of cyclin A1 and cyclin B1 protein (Figure 4C,D). Flow cytometry was used to detect cell apoptosis. The results showed that miR‐3647‐5p can significantly promote the apoptosis of Caski cells, while overexpression of AGR2 can reverse the effect of miR‐3647‐5p on Caski cells (Figure 4E,F). Western blot analysis also showed that AGR2 overexpression can reverse the effect of miR‐3647‐5p on Cyt C and Bax protein (Figure 4G). These results indicate that miR‐3647‐5p inhibits the proliferation of cervical cancer cells and promotes cell apoptosis by targeting AGR2.

### 3.5 TP53 acts as a transcription factor, up‐regulates miR‐3647‐5p, thereby inhibiting the expression of AGR2

**Figure 5:**
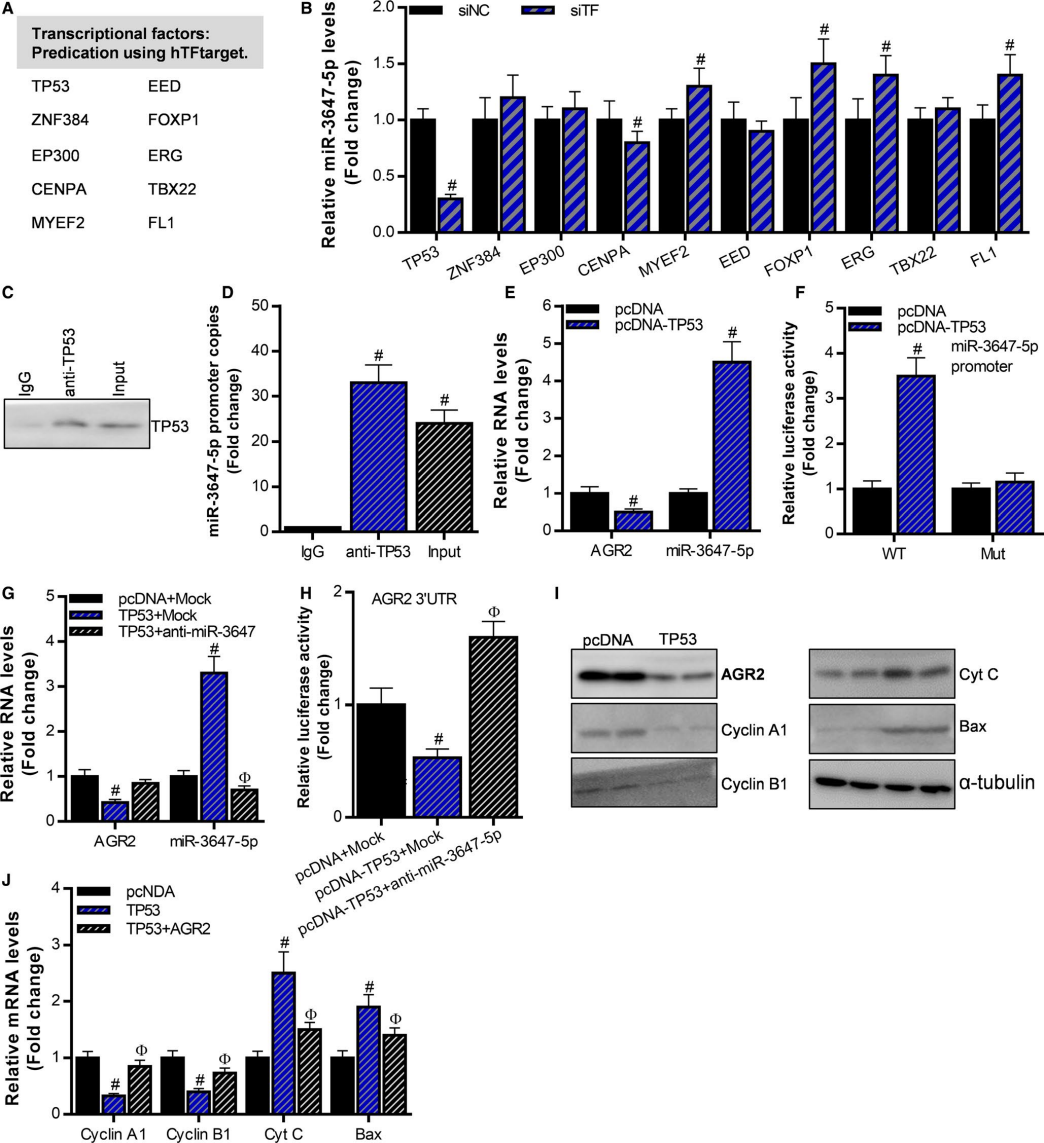
TP53 acts as a transcription factor, and up‐regulates miR‐3647‐5p, thereby inhibiting the expression of AGR2. A, Transcription factors that interact with miR‐3647‐5p are predicted using the hTFtarget database. B, The effect of transcription factor knockdown on the expression of miR‐3647‐5p was detected using reverse transcription and quantitative polymerase chain reaction (RT‐qPCR), ^#^ *P* < .05, compared to the Scramble group. (C, D) The interaction of TP53 with the miR‐3647‐5p promoter was detected using ChIP‐qPCR assay, ^#^ *P* < .05, relative to the immunoglobulin G group. E, Luciferase assay was used to detect the effect of TP53 overexpression on the activity of the miR‐3647‐5p promoter or binding site mutation reporter plasmids, ^#^ *P* < .05, relative to the pcDNA group. F, The effect of TP53 overexpression on the expression of AGR2 and miR‐3647‐5p was detected using RT‐qPCR, ^#^ *P* < .05, relative to the pcDNA group. G, The expression levels of AGR2 and miR‐3647‐5p in each group were detected using RT‐qPCR, ^#^ *P* < .05, relative to pcDNA + Mock group, ^Ф^ *P* < .05, relative to TP53 + Mock group. H, Luciferase assay was used to detect the activity of the AGR2 3'untranslated regions (3′UTR) reporter plasmid in each group of cells, ^#^ *P* < .05, relative to the pcDNA + Mock group, ^Ф^ *P* < .05, relative to the TP53 + Mock group. (I, J) Western blot was used to detect the effect of TP53 overexpression on AGR2, cell cycle‐related genes and apoptosis‐related genes, ^#^ *P* < .05, relative to pcDNA + Mock group, ^Ф^ *P* < .05, relative to TP53 + Mock group

In the present study, we confirmed that the expression of miR‐3647‐5p is down‐regulated during the development of cervical cancer, so what is the mechanism of dysfunction? We found that the promoter region of miR‐3647‐5p may be regulated by TP53, ZNF384, EP300, CENPA, MYEF2, EED, FOXP1, ERG, TBX22, FL1 at the transcriptional level (Figure 5A). In Caski cells, we knocked down these transcription factors by siRNA, and then detected the expression level of miR‐3647‐5p using RT‐qPCR. The results showed that knockdown of TP53 or CENPA can significantly inhibit the expression of miR‐3647‐5p, while knock down of MYEF2, FOXP1, ERG or FL1 significantly up‐regulated miR‐3647‐5p (Figure 5B). Among them, TP53 has the most obviously activated effect on miR‐3647‐5p. Next, we examined the interaction of the TP53 protein with the miR‐3647‐5p promoter using chromatin immunoprecipitation‐quantitative polymerase chain reaction (ChIP‐qPCR) assay, and the results showed that the TP53 interacted with the promoter of miR‐3647‐5p (Figure 5C,D). Using the luciferase assay, it was confirmed that TP53 significantly enhanced the activity of the miR‐3647‐5p promoter, but had no significant effect on the promoter in which the binding sequence was mutated (Figure 5E). In addition, overexpression of TP53 significantly up‐regulated the expression level of miR‐3647‐5p and inhibited AGR2 (Figure 5F). Anti‐miR‐3647‐5p reversed the effect of TP53 on miR‐3647‐5p and AGR2 expression (Figure 5G). Since TP53 up‐regulates the expression of miR‐3647‐5p, we also observed that TP53 overexpression inhibits the 3′UTR reporter gene activity of AGR2, whereas anti‐miR‐3647‐5p reverses the effect of TP53 on AGR2 (Figure 5H). Western blot analysis showed that TP53 overexpression inhibited the expression of AGR2, cyclin A1 and cyclin B1, and up‐regulated Cyt C and Bax (Figure 5I). In addition, overexpression of AGR2 reversed the effect of TP53 on cyclin A1, cyclin B1, Cyt C and Bax (Figure 5J). These results indicate that TP53 acts as a transcription factor, and up‐regulates miR‐3647‐5p, thereby inhibiting the expression of AGR2.

## 4 DISCUSSION

The mechanism of tumor development and development is multi‐level and multi‐factor. At the molecular level, it is mainly reflected in the dysfunction of oncogenes or tumor suppressor genes, resulting in imbalance of regulatory mechanisms of cell proliferation, migration, apoptosis and autophagy.^14^ The activation of oncogenes, or the inactivation of tumor suppressor genes, is mainly reflected in the dysfunction of their expression levels. Transcriptional and posttranscriptional levels are two important mechanisms regulating gene expression.^15^ In this study, we elucidated the important mechanism of TP53/miR‐3647‐5p/AGR2 axis in the development of cervical cancer. The mechanism included two aspects: at the transcriptional level, transcription factor TP53 up‐regulates the expression level of miR‐3647‐5p; at the posttranscriptional level, miR‐3647‐5p is able to target the 3′UTR of AGR2 mRNA, thereby inhibiting AGR2 expression.

P53, encoding by TP53 gene, is a famous and classical tumor suppressor which mainly acts as a transcription factor.^16^ When cells are under stress such as DNA damage, hypoxia, and oncogene activation, p53 is activated, which regulates a series of target genes, promoting cell cycle arrest, apoptosis, or aging.^17^ The p53 protein contains three domains, including the transcriptional activation domain at the N‐terminal acidic; the central sequence‐specific DNA binding domain; the C‐terminal basic region, including the nuclear localization signal region, homologous oligomerization zone, tetramerization domain and nuclear output signal zone.^18^ After entering the nucleus, p53 protein binds to the p53 response element of target genes through the DNA binding domain, or interacts with other transcription factors, activating the expression of target genes.^19^ A lot of target genes for p53 have been discovered, such as CDKN1A, mdm2, Bax. In this study, we found that tumor suppressor miR‐3647‐5p inhibits cell proliferation and promotes cell apoptosis in cervical cancer. Furthermore, we found that the promoter region of miR‐3647‐5p contains p53 response element. Through in vitro study, we have identified that miR‐3647‐5p is a target gene of p53 protein. In cervical cancer cells, overexpression of TP53 significantly up‐regulated the expression level of miR‐3647‐5p, suggesting that the dysfunction of miR‐3647‐5p in cervical cancer may be related to TP53.

3′untranslated regions play an important role in the mechanism of posttranscriptional regulation.^20^ They are involved in regulating the stability and degradation rate of mRNA, controlling the utilization efficiency of mRNA, and determining the translation site and translation efficiency of mRNA.^21^ miRNAs generally contain 2‐8 nucleotides complementary to the “seed region” of the 3′UTR of mRNA, blocking the translation of mRNA, or directly degrading the target mRNA, thereby transregulating the expression of the target gene.^22^ In this study, we demonstrated that miR‐3647‐5p is down‐regulated during the development of cervical cancer, and that overexpression of miR‐3647‐5p can inhibit the proliferation of cervical cancer cells and promote apoptosis, suggesting that miR‐3647‐5p may act as a tumor suppressor gene. It is known that MiRNAs primarily play a biological role by regulating the expression of target genes. Therefore, through bioinformatics analysis and molecular biology validation, we found that miR‐3647‐5p can target the 3′UTR of AGR2 and inhibit the expression of AGR2. AGR2 is a protein disulfide isomerase.^23^ Studies have found that compared to normal pancreatic tissues, the expression of AGR2 is up‐regulated in benign pancreatic lesions, low‐grade and high‐grade tumors.^24^, ^25^ In addition, some scholars have found that AGR2 is highly expressed in Barrett's esophageal metaplasia and esophageal adenocarcinoma.^26^, ^27^ In addition to precancerous lesions of pancreatic cancer and esophageal adenocarcinoma, AGR2 is also highly expressed in breast cancer, prostate cancer, ovarian cancer, and pancreatic cancer, and involved in the process of tumor cell proliferation and metastasis.^28^, ^29^, ^30^ These studies indicate that AGR2 acts as an oncogene and plays an important role in the progression of various malignancies. However, the correlation between AGR2 and cervical cancer is unclear. Through bioinformatics analysis, we found that AGR2 is highly expressed in cervical cancer tissue. Moreover, AGR2 overexpression can attenuate the inhibitory effect of miR‐3647‐5p on the proliferation of cervical cancer cells and the promotion of apoptosis, suggesting that AGR2 plays an oncogenic role in cervical cancer. It has been found that high expression of AGR2 is associated with down‐regulation of p53 in human tumor cell lines. But the mechanism is not clear. In our study, we also found that p53 inhibited the expression of AGR2. Further studies showed that p53 can inhibit the expression of AGR2 by up‐regulating miR‐3647‐5p. Our study shows that in cervical cancer cells, p53‐mediated miR‐3647‐5p inhibits cell proliferation and promotes cell apoptosis by targeting AGR2.

In conclusion, we found that miR‐3647‐5p is down‐regulated during the progression of cervical cancer, and explains the mechanism of TP53/miR‐3647‐5p/AGR2 axis in cervical cancer. At the transcriptional level, transcription factor TP53 up‐regulates the expression level of miR‐3647‐5p; at the posttranscriptional level, miR‐3647‐5p is able to target the 3′UTR of AGR2 mRNA, thereby inhibiting AGR2 expression. Our study reveals that TP53/miR‐3647‐5p/AGR2 axis inhibits the proliferation of cervical cancer cells and promotes apoptosis, providing a new strategy for targeted therapy of cervical cancer.

## CONFLICT OF INTEREST

All authors declare that there are no conflicts of interests.

## Data Availability

The data used to support the findings of this study are available from the corresponding author upon request.
